# Editorial: Clinical, health services and legal dimensions in older adult care: a focus on the Middle Eastern and Northern African region

**DOI:** 10.3389/fpubh.2026.2007553

**Published:** 2026-10-28

**Authors:** Madhan Balasubramanian, Hossein Esmaeili, Mohammed Khaled Al-Hanawi

**Affiliations:** 1 Flinders Business School and Centre for Social Impact, Flinders University, Adelaide, SA, Australia; 2 School of Public Health and Leeder Centre for Health Policy, Economics and Data, University of Sydney, Sydney, NSW, Australia; 3 Flinders Law School, Flinders University, Adelaide, SA, Australia; 4 Health Services and Hospitals Administration Department, Faculty of Economics and Administration, King Abdulaziz University, Jeddah, Saudi Arabia

**Keywords:** aged care, clinical, health services, legal, MENA region (Middle East and North Africa), models of care

## Introduction

The Middle East and North Africa (MENA) region is undergoing a rapid demographic transition, placing new demands on health care and its regulatory and policy systems. Globally, the population aged 60 and over is projected to nearly double, from 12% in 2015 to 22% by 2050 (1), and the Eastern Mediterranean Region is aging from a younger base and at a faster relative pace (2). Saudi Arabia's older population is projected to more than triple as a share of the total by 2050 (Alsaeed et al.), and Morocco's cancer burden is rising in step with its own transition (Sabri et al.). Health systems, workforces, and financing models built around a younger population profile are being asked to meet the needs of a rapidly growing older population. This Research Topic gathered clinical, health services, management, and regulatory and policy scholarship addressing that shift. Its eight published items (seven original studies and one correction), drawn largely from Saudi Arabia with contributions from Qatar and Morocco, provide insight into the current state of evidence on older-adult care in parts of the MENA region and point to important directions for future research, policy, and practice.

## A collection organized around five practical questions

The collection clusters around five questions any aged-care system must eventually answer: can disease be detected early enough to intervene; can people access and afford the care they need; is care delivered where and how older patients can use it; does the informal, family-based system that carries much of the load continue to hold as cohorts grow; and is policy governance capable of steering the response rather than simply reacting to it. Each article speaks most directly to one or two of these questions, and together they begin to build a fuller picture across all five. Table 1 summarizes the seven studies.

**Table 1:** Key findings from the seven studies in this Research Topic.

#	Study and Country	Design/sample	Key finding	Policy/practice implication
1	Alnuaimi et al. —AF screening in primary care Qatar	Cross-sectional pilot study; *n* = 249, aged ≥65	AF prevalence 5.4%; 2% newly diagnosed, all meeting anticoagulation criteria (CHA_2_DS_2_-VA). Family physicians matched cardiologists on 75% of ECG reads. Scaled nationally, could surface ~747 undiagnosed cases among 37,371 individuals aged ≥65.	A primary-care-led, age-targeted (≥75) ECG screening model is feasible and may reduce costs-effective for stroke prevention in Qatar; supports task-shifting AF detection to family physicians.
2	Alsaeed et al. —Older adult care landscape (systematic review) Saudi Arabia	PRISMA-guided systematic review; English + Arabic sources, 1995–2025	System “evolving but under strain”: measurable progress on long-term care and hospital-based geriatric programs under Vision 2030, but workforce shortages, heavy reliance on migrant caregivers, digital illiteracy, and fragmented evidence base persist.	Reform momentum needs to be matched with workforce investment and a national aging-research agenda to close the evidence gap.
3	Sabri et al. —Oncogeriatric transition (narrative review) Morocco	Structured narrative review; policy/registry/literature sources, 2000–2025	Adults ≥60 = 13.8% of the population (2024); registries show high vulnerability (>80% abnormal G8 in some cohorts) and frequent metastatic presentation; structured geriatric assessment not yet routine in oncology care. Proposes a Four-Phase Oncogeriatric Transition framework.	Cancer control strategy needs to integrate geriatric assessment and workforce capacity-building now, ahead of the demographic curve, rather than retrofitting later.
4	Alanazi et al. —Telehealth adoption among neurologists Saudi Arabia	National cross-sectional survey; *n* = 300 neurologists	78.7% intend to use telehealth; confidence far higher for follow-up care (82.7%) than new-patient consults (24.0%). Familiarity with telehealth was the strongest independent predictor of adoption intent (OR = 32.0).	Digital-health scale-up for neurology (relevant to dementia/stroke follow-up in older patients) should prioritize clinician familiarization and audio-service infrastructure over new-patient use cases first.
5	Moubarak et al. —Social health indicators and quality of life (+ published correction) Saudi Arabia	Descriptive study using the Social Health Scale for Older People; *n* = 660, aged ≥65	High social-support (*M* = 2.63) and social-adjustment (*M* = 2.41) scores, attributed to family-centered, culturally-rooted care norms; study flags this informal model is under pressure from projected demographic growth.	Family/community-based care remains a genuine strength to build on, but needs formal policy backing (community initiatives, institutional-care access) before demographic pressure erodes it.
6	Hariri et al. —Evolution of health policymaking Saudi Arabia	Qualitative study; thematic analysis of 10 semi-structured expert interviews (NVivo)	Shift from centralized to collaborative, evidence-based policymaking; key gaps: weak policy governance and no systematic evaluation process. Study calls for reduced reliance on foreign policy expertise and a centralized policy repository.	Aging policy will only be as good as the governance and evaluation infrastructure behind it—a repository and local policy-capacity building are prerequisite investments.
7	Al-Hanawi and Keetile —Out-of-pocket expenditure on medicines Saudi Arabia	Cross-sectional analysis of the 2018 Family Health Survey; *n* = 10,785; Tobit regression	Mean OOP medicine spend = SAR 279.69; driven up by age, income, education, and chronic disease; married/employed status associated with lower OOP spend. Flags catastrophic-expenditure risk for the most vulnerable.	Financial protection mechanisms (insurance/subsidy design) for medicines need to be age- and chronic-disease-targeted, not just universal, to prevent catastrophic health expenditure among older adults.

This Research Topic comprises eight published items—seven original studies and one formal correction to the Moubarak et al., which does not alter the reported findings and is treated here as part of the same study rather than a separate row. The seven studies are summarized above.

**On detection**, Alnuaimi et al. screened 249 adults aged 65 and over in Qatari primary care and found an atrial fibrillation prevalence of 5.4%, with 2% newly diagnosed; family physicians matched cardiologists on 75% of diagnoses, suggesting stroke prevention can advance through task-shifting rather than new infrastructure; scaled nationally, the authors estimate roughly 747 undiagnosed cases could be found among those aged 65 and over. **On affordability**, Al-Hanawi and Keetile, analyzing 10,785 respondents, showed that out-of-pocket medicine expenditure in Saudi Arabia (mean SAR 279.69) rises with age, income, and chronic illness, so financial exposure persists under free public care and concentrates on those least able to absorb it, a catastrophic-expenditure risk calling for targeted insurance and subsidy design. **On delivery**, Alanazi et al. found that 78.7% of Saudi neurologists intended to use telehealth, with far greater confidence for follow-up (82.7%) than for new patients (24.0%) and familiarity the strongest predictor of intent (odds ratio 32.0), a sequencing lesson for digital-health rollouts in conditions such as dementia and stroke. **On the informal system**, Moubarak et al. documented strong family-centered social support among older Saudis (high social-support and social-adjustment scores, attributed to family-centered, culturally rooted care norms) while cautioning that it will be tested as cohorts grow and recommending formal community-based capacity now; shared-responsibility community care models suggest formal and informal care can reinforce rather than compete with each other (3). **On governance**, Hariri et al. traced a shift toward more collaborative, evidence-informed policymaking in Saudi Arabia, alongside weak evaluation structures and reliance on external expertise, and recommended a centralized policy repository with investment in local policy capacity; given the study's exploratory design and small qualitative sample, these findings warrant validation in future research.

Two contributions provide scaffolding for the field. Alsaeed et al. conclude in their systematic review that Saudi Arabia's older adult care system is “evolving but under strain”, with progress under Vision 2030 set against workforce shortages and reliance on migrant caregivers. Sabri et al. propose a Four-Phase Oncogeriatric Transition framework for Morocco, where over 80% of some cohorts screened abnormal on the G8 vulnerability tool and geriatric assessment is not yet routine in oncology care. Both suggest that studies in the region are accumulating faster than the frameworks needed to interpret and act on them.

## Toward a regional framework

Taken together, these studies give the region a working foundation of methods and findings that can be extended across MENA health systems, given similarities in cultural, regulatory, and policy contexts. Opportunities include carrying the primary-care screening model of Alnuaimi et al. to other Gulf and MENA systems, and testing the emerging frameworks of Alsaeed et al. and Sabri et al. against data from other North African and Levant countries. The Research Topic invited clinical, health services, and legal perspectives together; this collection has built strongly on the first two, and now offers a starting point from which the third, along with business, social work, and industry voices, can be drawn in as the field's next chapter.

The comparatively limited representation of legal and regulatory scholarship in the present collection also identifies an important gap. Future work should examine regulatory frameworks for long-term care, rights and protection of older persons, consent and decision-making capacity, governance of digital technologies, regulation of formal and informal caregiving, and accountability for quality and safety in aged-care services. Aging systems research more broadly benefits from treating workforce, models of care, technology, governance, financing, and place-based environments as elements of a single system (4).

## Capacity-building efforts under way

Bringing these additional disciplines into the conversation is also the kind of work that regional capacity-building initiatives are beginning to take on, in parallel with this Research Topic rather than produced by it (Figure 1). One example connected to members of the editorial team is the MENA Hub for Business, Law, Regulation, Health and Aged Care, now forming through the Australian–Arab Collaborative at Flinders University with support from a DFAT Council for Australian–Arab Relations grant. It seeks to bring legal and regulatory scholars, business specialists, and health and aged-care researchers into closer contact with government, university, and industry partners, beginning with a two-part symposium series held across Adelaide, Cairo, and Jeddah and a partnership between Flinders University and King Abdulaziz University. It sits alongside the Flinders Ageing Alliance, which connects research teams in Adelaide and Jeddah with collaborators across more than 10 countries, including Egypt, Qatar, the United Arab Emirates, India, Nepal, Bangladesh, Malaysia, Indonesia, China, Singapore, and the Philippines (5). An earlier DFAT-funded fellowship that brought 15 aged-care leaders from six Asia-Pacific nations to Australia in 2023 produced its own special issue in this journal (6) and a supplement in Health Care Management Review that included a scoping review of long-term care policies across the participating countries (7), showing how a fellowship network can convert into published regional research within a single funding cycle; the progress of the MENA Hub will be a matter for future evaluation.

**Figure 1:**
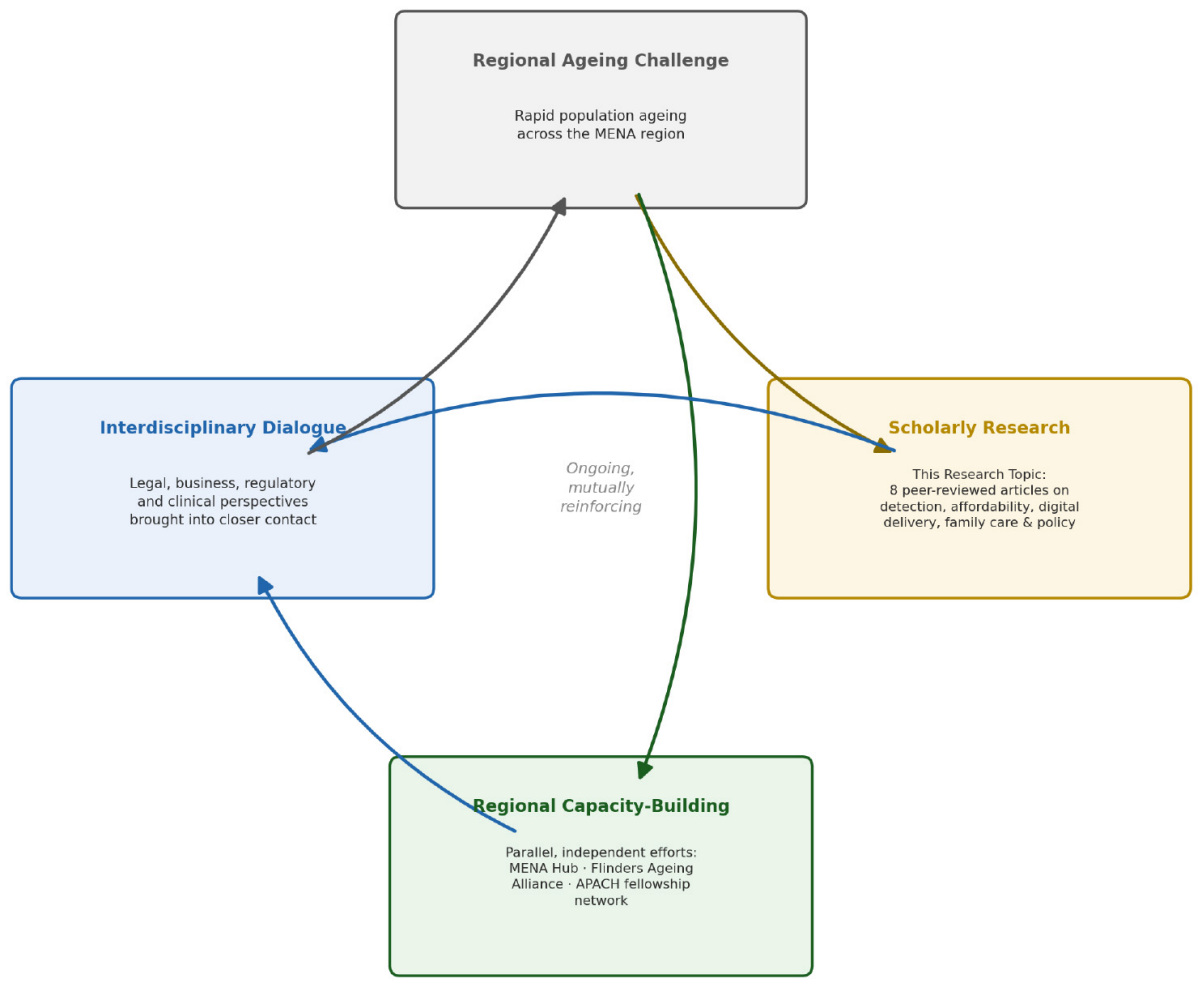
A cycle of regional response, not a single pipeline. This Research Topic and regional capacity-building efforts (the MENA Hub, the Flinders Ageing Alliance, and the APACH Asia-Pacific fellowship network) are independent, parallel responses to the same regional aging challenge. Both feed into a broader interdisciplinary dialogue that shapes how the region continues to respond.

## Looking ahead

Taken together, the articles in this Research Topic make an important, though necessarily partial, contribution to the emerging evidence base on older-adult care in the MENA region. We hope this collection marks the beginning of an ongoing series of contributions. The next phase will require comparative, interdisciplinary and policy-oriented research capable of translating demographic change into evidence-informed approaches to prevention, financing, service delivery, workforce development, regulation, and protection of older adults across diverse MENA health systems.
